# Impact of the COVID-19 and psychological risk factors on non-suicidal self-injury behavior among high school students: a one-year follow-up study

**DOI:** 10.1186/s12888-023-05021-2

**Published:** 2023-07-14

**Authors:** Lu-Jiao-Zi Wang, Yan Lan, Su-Jiao Liu, Wan-Sen Yan

**Affiliations:** 1 Department of Psychology, School of Medical Humanitarians, Guizhou Medical University, 9 Beijing Road, Yunyan District, Guiyang, 550004 China; 2 Guizhou Research Institute for Health Development, Guizhou Medical University, Guiyang, China

**Keywords:** Non-suicidal self-injury, Adolescent, Students, Risk factor, COVID-19

## Abstract

**Objectives:**

Non-suicidal self-injury (NSSI) behavior is a severe public health issue in adolescents. This study investigated the possible impact of the coronavirus disease 2019 (COVID-19) and analyzed psychological risk factors on adolescent NSSI.

**Methods:**

A one-year follow-up study was conducted in September 2019 (Time 1) and September 2020 (Time 2) among 3588 high school students. The completed follow-up participants (*N* = 2527) were classified into no NSSI (negative at both time points), emerging NSSI (negative at Time 1 but positive at Time 2), and sustained NSSI (positive at both time points) subgroups according to their NSSI behaviors before and during the COVID-19 pandemic. Perceived family functioning, perceived school climate, negative life events, personality traits (neuroticism, impulsivity, and self-control) were assessed using self-report scales.

**Results:**

The data indicated an increase (10.3%) in the incidence of NSSI. Compared to no NSSI subjects, the emerging NSSI and sustained NSSI subgroups had lower perceived family functioning, higher neuroticism, higher impulse-system but lower self-control scores, and more negative life events. Logistic regressions revealed that after controlling for demographics, neuroticism and impulse-system levels at Time 1 positively predicted emerging NSSI behavior, and similarly, higher neuroticism and impulsivity and lower self-control at Time 1 predicted sustained NSSI behavior.

**Conclusions:**

These findings highlighted the aggravated impact of the COVID-19 on NSSI, and suggested that individual neuroticism, impulsivity, and self-control traits might be crucial for the development of NSSI behavior among adolescent students.

**Supplementary Information:**

The online version contains supplementary material available at 10.1186/s12888-023-05021-2.

## Introduction

The outbreak of the coronavirus disease 2019 (COVID-19) was declared as a pandemic by the World Health Organization (WHO) in March 2020. Prevention strategies such as social distancing, quarantine, and lockdown during the pandemic have brought about mental health issues in general populations, including depression, anxiety, self-harm, suicide ideation and attempt [1–4]. Particularly, these symptoms would be more likely to occur among children and adolescents, mainly due to their psychological and developmental vulnerabilities associated with immaturity [5, 6]. For this reason, call-to-action for longitudinal mental health research during (and after) the COVID-19 pandemic has been raised to understand the long-term impact of the pandemic on children and adolescents [7, 8]. Although systematic reviews suggested a deterioration in mental health among children and youths from before to during the pandemic, there was little direct evidence from longitudinal studies [9, 10]. The present study thus aimed to investigate the impact of the pandemic on adolescent non-suicidal self-injury (NSSI) and examine psychological risk factors.

NSSI is defined as the self-directed deliberate destruction or alteration of one’s own body tissue without suicidal intent [11, 12]. NSSI behavior is a growing public health problem with an estimated lifetime prevalence of 4–6% in adult community samples [13, 14], and is most common among adolescents [15, 16], with the global prevalence estimated to be about 22.0% in a lifetime period and 23.2% during a 12-month period [17]. There is mounting evidence of cross-sectional studies on NSSI incidence and related risk factors among school-based adolescents and young adults in China [18–24], while longitudinal research remains scarce [25]. The COVID-19 pandemic and its transmission mitigation measures have lead to a rise in self-harm and NSSI [26], especially for adolescents and school students [27–29]. Generally, in the Chinese cultural context, senior high school students are having great pressure from the fierce competition and the high expectation of their success from parents in the national college entrance examination. Therefore, it should be of interest to understand the development of NSSI behavior in these students before and during the pandemic. Moreover, an investigation into the key psychological variables underpinning the occurrence and continuation of NSSI could be helpful for potential development of prevention and intervention measures for adolescent NSSI.

From the theoretical person-environment framework of risk factors for NSSI [30–33], the individual, familial, and social contributors to NSSI behaviors may probably include personality traits such as impulsivity [34, 35], family functioning and structure [36, 37], social/peer support or rejection [38–41], as well as stressful life events [42, 43]. Notably, these complex factors might not separately play a part in the development of adolescent NSSI, but rather work together as the cumulative effects [44, 45]. Based on these theoretical points, the current study concurrently considered multiple risk factors in the longitudinal trajectories of NSSI among high school students, including perceived family functioning and school climate, negative life events, as well as personality traits (neuroticism, impulsivity, and self-control).

In the first place, family plays an important role in adolescents’ mental growth and provides the main spiritual and material support system [46, 47]. Poor family functioning (e.g., deficient emotional bonding between the family members) has been found to be positively associated with more NSSI behaviors [48–50], and family dysfunction was indicated as a robust risk factor for NSSI in adolescents [51, 52]. As a whole, family functioning is an important indicator of family structure that may contribute to the development of adolescent NSSI [36]. Relatively speaking, perceived school climate has been less studied in the literature of NSSI. Several studies revealed that school-related factors including teachers’ support and peer climate were negatively associated with NSSI in high school students [41, 53], and school bullying and peer rejection were closely related to adolescent NSSI [38, 39]. It was supposed that students would be less likely to engage in NSSI when feeling supported by their teachers and peers within a positive school climate [41], while more attention should be paid to the role of perceived school climate in NSSI [54]. Stressful or negative life events have been proved to be harmful for the development of mental health in adolescents [55, 56]. Positive relationships between perceived life stress and NSSI have also been indicated among adolescent and college students [43, 57, 58], despite that frequent use of cross-sectional analyses might leave unclear the precise nature of the relation between life stress and NSSI [42]. The COVID-19 pandemic represents a unique challenge for individuals, and pandemic-related stress may increase the risk for self-injurious behaviors in adolescents [6, 59]. Especially, pre-existing vulnerabilities such as poor regulatory emotional self-efficacy and higher levels of internalizing symptoms before the pandemic might increase the risk to engage in NSSI for adolescents [59]. However, the effects of family functioning, school climate, and life stress on the continuation and development of NSSI in high school students remain to be further understood before and during the pandemic.

On the other side, certain typical personality traits such as neuroticism, impulsivity and self-control, have been linked to various maladaptive behaviors and mental disorders, as the potential individual vulnerabilities [60–63]. Notably, higher levels of neuroticism were associated with deliberate self-harm and NSSI [64–66], and neuroticism was a positive predictor for NSSI in adolescent and college students [67]. Despite the limited evidence, heightened impulsivity assessed by self-report measures was revealed in individuals with NSSI [34, 68], and laboratory task-based behavioral impulsivity was also associated with NSSI behaviors [35, 69]. Conversely, poor self-control abilities, impulse control difficulties, and self-regulatory difficulties constituted the risk factors for adolescent NSSI [70–72]. These individual traits could also be crucial for the development of NSSI behaviors in the context of pandemic.

Considering the above-mentioned issues, the purpose of the present study was (i) to investigate the possible impact of the COVID-19 pandemic on the continuation and development of NSSI, and (ii) to test the longitudinal links between NSSI and perceived family functioning and school climate, perceived life stress, as well as personality traits (i.e., neuroticism, impulsivity, and self-control), using a follow-up study design in a large sample of adolescent high school students. We generally hypothesized that the multiple individual and environmental factors might take effect collectively in the development of adolescent NSSI during the COVID-19 pandemic.

## Methods

### Participants and procedure

As part of a longitudinal follow-up study on NSSI among adolescents, a total of 3588 senior high school students were randomly selected in September 2019 (Time 1) from the nine main cities of Guizhou Province, which is located in the southwest of China. Firstly, a summary list of senior high schools in each city was created, and a total of 24 high schools, accounting for about 10% of the whole, were then randomly selected with 2 or 3 high schools in each city. Next, the survey was conducted in these schools by well-trained psychology teachers and graduate students. In each school, one random class in each grade (i.e., 10th, 11th, and 12th) was chosen, and students in these classes were invited to participate in this study. Instructions for the study purposes, methods, and procedure were clearly printed on a sheet of paper, along with the written informed consent. The students could decide freely whether to be enrolled or not, after consulting with their legal guardian at home. After the students gave the written informed consent signed and provided by their legal guardian, a series of self-report scales were completed independently by students in a 45-minute group activity class. Those students who had a self-reported history of psychoactive substance use or abuse, psychiatric disorders, neurological diseases, brain trauma, or severe physical diseases, were excluded from the study enrollment.

In September 2020 (Time 2) during the COVID-19 pandemic, the prior 3588 participants were invited again to participate in research, through telephone, WeChat, or QQ (an instant messaging program). Of all participants, 1061 individuals failed to respond due to lost contact after graduation or under disease-related quarantine. A total of 2527 subjects (70%) effectively responded to our invitation, who gave the written informed consent provided by their legal guardian online, and completed the same series of self-report scales as those in Time 1 (but in a different randomized order), through the online survey programming platform (i.e., WenJuanXing). Demographic characteristics between the 2527 subjects and prior 3588 participants were compared to test the sample homogeneity (please see Supplementary Table S1). There were no significant differences on gender, ethnicity, home locality, proportion of single-child, proportion of left-behind child, and education level of father/mother, except for age, between these two samples. Therefore, data of the 2527 participants, as the completed follow-up sample, were reported and analyzed in the current study.

According to the NSSI scores evaluated by the Functional Assessment of Self Mutilation tool [73, 74], 686 subjects (27.2%) at Time 1 and 947 subjects (37.5%) at Time 2 were identified as individuals with NSSI in the follow-up sample (*N =* 2527). The demographic characteristics of the follow-up sample were displayed for the two time points in the Supplementary Table S2. Briefly, this sample had an average age of 16.13 ± 0.79 years (ranging from 15 to 18 years) at Time 1, with 1217 boys (48.2%).

For the purpose of investigating the impact of the COVID-19 pandemic and the effects of psychological risk factors associated with NSSI, we classified all subjects into no NSSI (i.e., negative at both time points), emerging NSSI (i.e., negative at Time 1 but positive at Time 2), sustained NSSI (i.e., positive at both time points), and recovered (i.e., positive at Time 1 but negative at Time 2) subgroups, according to their NSSI behavior before and during the pandemic. Accordingly, there were 1580 individuals (62.5%) in no NSSI subgroup, 261 (10.3%) in emerging NSSI subgroup, and 686 (27.2%) in sustained NSSI subgroup, bur zero for the recovered subgroup. As a result, further analyses were based on the former three subgroups (Table 1).

**Table 1:** Demographic characteristics of subgroups in the follow-up sample (*N* = 2527)

Variables	a. No NSSI	b. Emerging NSSI	c. Sustained NSSI	*F*/χ^2^	Post-hoc test (*p* < 0.05)
Number of subjects, n (%)	1580 (62.5)	261 (10.3)	686 (27.2)	-	-
Years of age (M ± SD), T1	16.19 *±* 0.79	15.96 *±* 0.79	16.07 *±* 0.78	13.065***	a > b = c
Years of age (M ± SD), T2	17.19 *±* 0.79	16.96 *±* 0.79	17.07 *±* 0.78	13.065***	a > b = c
Gender, Boys n (%) ^a^	766 (48.5)	122 (46.7)	329 (48.0)	0.286	-
Ethnicity, Hans n (%) ^a^	1404 (88.9)	229 (87.7)	587 (85.6)	4.861	-
Single-child, Yes n (%) ^a^	251 (15.9)	28 (10.7)	78 (11.4)	10.813**	a > b = c
Left-behind child, Yes n (%) ^a^	570 (36.1)	122 (46.7)	293 (42.7)	16.231***	a < c < b
Home locality, Urban n (%) ^a^	1009 (63.9)	103 (39.5)	249 (36.3)	170.499***	a > b = c
Grade, n (%), T1				48.775***	
10th Grade	717 (45.4)	171 (65.5)	385 (56.1)		a < c < b
11th Grade	863 (54.6)	90 (34.5)	301 (43.9)		a > c > b
12th Grade	-	-	-		-
Grade, n (%), T2				48.775***	
10th Grade	-	-	-		-
11th Grade	717 (45.4)	171 (65.5)	385 (56.1)		a < c < b
12th Grade	863 (54.6)	90 (34.5)	301 (43.9)		a > c > b
Education level of father, n (%) ^a^				60.494***	
Junior high school and below	909 (57.5)	181 (69.4)	507 (73.9)		a < b < c
Senior high school	494 (31.3)	58 (22.2)	126 (18.4)		a > b > c
College/university and above	177 (11.2)	22 (8.4)	53 (7.7)		a > b = c
Education level of mother, n (%) ^a^				112.614***	
Junior high school and below	989 (62.6)	207 (79.3)	572 (83.4)		a < b < c
Senior high school	352 (22.3)	31 (11.9)	78 (11.4)		a > b = c
College/university and above	239 (15.1)	23 (8.8)	36 (5.2)		a > b > c
NSSI/FASM scores (M ± SD), T1	0.00 *±* 0.00	0.00 *±* 0.00	10.79 *±* 7.42	-	-
NSSI/FASM scores (M ± SD), T2	0.00 *±* 0.00	5.51 *±* 4.20	13.55 *±* 8.29	2155.716***	a < b < c

Note: NSSI = Non-suicidal self-injury. FASM = the Functional Assessment of Self-Mutilation.

No NSSI = the subgroup of individuals without NSSI (i.e., who were negative in both time points)

Emerging NSSI = the subgroup of individuals who were negative in Time 1 but positive in Time 2

Sustained NSSI = the subgroup of individuals who were positive in both time points

T1 = Time 1 (Sep. 2019), T2 = Time 2 (Sep. 2020)

^a^ The variable had a same value each at T1 and T2

** *p* < 0.01, *** *p* < 0.001

### Measures

#### Functional assessment of self mutilation (FASM)

We used the standard FASM tool [73, 74] to assess NSSI in adolescents. The FASM is a self-administered checklist consisting of 12 types of NSSI (i.e., hitting self, biting self, pulling hair, inserting objects under nails or skin, picking at a wound, picking areas to draw blood, cutting or carving, burning, self-tattooing, scraping, erasing skin, and other category). Subjects were asked whether they purposefully engaged in each NSSI behavior within the past year and, if so, the frequency of occurrence. Subjects with a zero score on the FASM were considered negative individuals without NSSI behavior, while those with a score of 1 or more were considered positive individuals with NSSI behavior. In addition to this dichotomy of negative or positive NSSI, the total FASM score was also used as a continuous variable and considered a proxy for severity of NSSI behavior. In this study, the Cronbach’s α of the FASM was 0.824 (Time 1) and 0.813 (Time 2), respectively.

#### Family adaptability and cohesion scale (FACES II)

The FACES II [75] is a 30-item scale that assesses perceived family functioning (i.e., family adaptability and family cohesion) on a 5-point Likert scale from 1 (not at all true) to 5 (always true). Specifically, family adaptability refers to the ability of a family to change in response to developmental or situational stress, while family cohesion refers to the degree to which the family members are connected with each others (i.e., the emotional bonding between family members). A higher score on the scale represents greater perceived family functioning. Family adaptability and family cohesion are analyzed separately as two dimensions. The Cronbach’s α values were 0.863 (Time 1) and 0.861 (Time 2) for family adaptability, and 0.852 (Time 1) and 0.850 (Time 2) for family cohesion, respectively.

#### School climate scale (SCS)

The SCS is a perceived school climate measure that assesses three dimensions of school climate, including teacher support, peer support (student-student support), and opportunities for autonomy in the classroom [76]. This scale consists of 25 items rated on a 4-point response scale (1 = never, 4 = always). Some item examples are such as “My teachers care about me” (teacher support), “Students trust one another” (peer support), and “Students have a say in how things work” (autonomy opportunities).

Higher scores on the SCS indicate higher levels of perceived teacher/peer support or autonomy opportunities. The Cronbach’s α values were 0.773 (Time 1) and 0.769 (Time 2) for teacher support, 0.794 (Time 1) and 0.792 (Time 2) for peer support, and 0.654 (Time 1) and 0.651 (Time 2) for autonomy opportunities, respectively.

#### Adolescent self-rating life events checklist (ASLEC)

The ASLEC was used to evaluate subjective suffering from negative life events experienced during the past 12 months [47]. It consists of 26 life events, using a 6-point response scale ranging from 0 (did not occur) to 5 (occurred and extremely stressful). Five types of negative life events are listed in the scale (i.e., interpersonal problems such as experiencing discrimination by others, school-related problems such as having a heavy workload, parental problems such as death of a parent, health and adaptation problems such as having severe illness, punishment and loss such as suffering a loss by theft). A higher total score represents more stressful life events experienced. The Cronbach’s α was 0.914 (Time 1) and 0.920 (Time 2), respectively.

#### Eysenck personality questionnaire-revised short form (EPQR-S)

The EPQR-S assesses three dimensions of individual personality (neuroticism, extraversion, psychoticism) with 12 items for each dimension, using yes/no response options (yes = 1, no = 0) [77]. Higher scores indicate higher levels of personality traits. In this study, we only tested the levels of neuroticism, using the EPQR-S neuroticism subscale. Because neuroticism represents the degree of emotionality for one subject, and is characterized by high levels of negative emotions (e.g., depression and anxiety) especially in response to unexpected stress, then the possible links of neuroticism with NSSI behavior before and after the COVID-19 would be of interest in this study. The Cronbach’s α was 0.889 (Time 1) and 0.892 (Time 2), respectively.

#### Dual-modes of self-control scale (DMSC)

The DMSC is a self-report measure for assessing the dual-system processes of behavioral control (i.e., impulse system and self-control system) [78, 79]. This scale consists of 21 items (12 items for impulse system and 9 items for self-control system) rated on a 5-point Likert scale from 1 (not at all true) to 5 (always true). The dimension of impulse system includes three factors (i.e., impulsiveness, easy distraction, and inability to delay gratification), and the dimension of self-control system includes two factors (i.e., problem solving and future-oriented time view). Higher scores indicate a higher level of impulse system or self-control system. Some item examples are such as “ I often act without careful thinking”, “I don’t pay attention to lectures”, and “I can’t save money but spend money quickly” (impulse system), and “I like thinking about and solving complex problems” and “I am future oriented” (self-control system). In this study, the Cronbach’s α values were 0.933 (Time 1) and 0.916 (Time 2) for the dimension of impulse system, and 0.887 (Time 1) and 0.875 (Time 2) for the dimension of self-control system, respectively.

### Statistical analysis

The data were analyzed with the Statistical Package for the Social Sciences for Windows, Version 22.0 (SPSS Inc., Chicago, IL, USA). Categorical variables (e.g., gender, ethnicity, grade, home locality, single-child, left-behind child, and education level of father/mother) were analyzed with chi-square tests for group comparisons. Psychological variables were compared between groups using multivariate analysis of variance (mANOVA) models, with demographic variables that were significantly different between the three subgroups as covariates (i.e., age, grade, home locality, single-child, left-behind child, and education level of father/mother). Post-hoc tests were conducted with Fisher’s least significant differences protected *t*-test, using Bonferroni correction for multiple comparisons. Relationship between psychological variables and NSSI/FASM scores was tested using partial correlations, controlling for all demographic variables. Logistic regression models were used to test the effects of perceived family functioning, perceived school climate, negative life events, neuroticism, impulse system and self-control system on emerging NSSI and sustained NSSI behaviors, controlling for all the demographics. According to the variance inflation factor (VIF), multicollinearity was not a problem for any variable (VIF < 10). The threshold for statistical significance was set at *p* < 0.05, two-tailed.

## Results

### Demographic characteristics of the subgroups

As presented in Table 1, there were no significant group differences observed for gender or ethnicity between the no NSSI, emerging NSSI, and sustained NSSI groups. However, the no NSSI group was slightly older than the other two groups (*p* < 0.001). There was a lower rate of single-child in the emerging NSSI (10.7%) and sustained NSSI (11.4%) groups compared with the no NSSI group (15.9%) (*p* < 0.001). The percentage of left-behind child was highest in the emerging NSSI group (46.7%), followed by the sustained NSSI group (42.7%), and lowest in the no NSSI group (36.1%) (*p* < 0.001). There was a higher rate of students from urban areas in the no NSSI group (63.9%) than the other two groups (39.5% and 36.3%) (*p* < 0.001). On the parents’ educational level, the no NSSI group was higher than the other two groups, with a higher rate of parents’ education of senior high school and above (*p* < 0.001).

### Group differences in psychological variables

Scores on the psychological variables were displayed for subgroups in Table 2.

**Table 2:** Psychological variables for the three subgroups (M ± SD).

Variables	a. No NSSI (n = 1580)	b. Emerging NSSI (n = 261)	c. Sustained NSSI (n = 686)	*F*	Post-hoc test (*p* < 0.05)
**Family functioning (FACES)**					
Family adaptability (T1)	46.32 ± 8.13	43.75 ± 9.05	38.30 ± 9.59	205.706***	a > b > c
Family adaptability (T2)	44.83 ± 8.03	42.31 ± 8.99	37.95 ± 9.51	155.033***	a > b > c
Family cohesion (T1)	54.74 ± 9.11	51.93 ± 9.69	46.18 ± 10.31	193.912***	a > b > c
Family cohesion (T2)	52.97 ± 9.35	50.72 ± 9.57	45.12 ± 10.20	159.806***	a > b > c
**Perceived school climate (SCS)**					
Teacher support (T1)	17.73 ± 3.49	17.41 ± 3.83	16.30 ± 3.66	38.453***	a = b > c
Teacher support (T2)	15.63 ± 3.47	15.23 ± 3.44	14.46 ± 3.52	27.303***	a = b > c
Peer support (T1)	41.97 ± 5.32	41.44 ± 5.93	38.57 ± 5.24	97.052***	a = b > c
Peer support (T2)	38.37 ± 6.03	37.95 ± 6.43	35.48 ± 5.25	58.875***	a = b > c
Autonomy opportunities (T1)	10.76 ± 2.69	10.67 ± 2.48	10.60 ± 3.07	0.851	-
Autonomy opportunities (T2)	11.74 ± 2.68	11.70 ± 2.38	11.62 ± 3.24	0.454	-
**Negative Life events (ASLEC)**					
Total life events (T1)	21.55 ± 11.28	22.90 ± 13.37	42.42 ± 18.52	560.261***	a = b < c
Total life events (T2)	26.47 ± 14.40	40.33 ± 19.78	46.85 ± 19.35	391.007***	a < b < c
**Personality trait (EPQ)**					
Neuroticism (T1)	2.72 ± 2.47	5.35 ± 3.44	8.21 ± 3.19	940.360***	a < b < c
Neuroticism (T2)	2.90 ± 2.65	4.87 ± 3.26	8.36 ± 3.11	878.364***	a < b < c
**Dual-system processes (DMSC)**					
Impulse system (T1)	24.19 ± 7.05	30.14 ± 10.01	38.98 ± 10.93	712.121***	a < b < c
Impulse system (T2)	26.29 ± 7.00	29.75 ± 8.36	40.12 ± 10.76	666.356***	a < b < c
Self-control system (T1)	33.83 ± 4.87	31.86 ± 5.78	24.59 ± 7.36	622.033***	a > b > c
Self-control system (T2)	31.61 ± 4.85	29.77 ± 5.60	23.14 ± 7.55	515.244***	a > b > c

Note: NSSI = Non-suicidal self-injury. No NSSI = the subgroup of individuals without NSSI (i.e., who were negative in both time points). Emerging NSSI = the subgroup of individuals who were negative in Time 1 but positive in Time 2. Sustained NSSI = the subgroup of individuals who were positive in both time points. T1 = Time 1 (Sep. 2019), T2 = Time 2 (Sep. 2020). FACES = Family Adaptability and Cohesion Scale II. SCS = School Climate Scale. ASLEC = Adolescent Self-Rating Life Events Checklist. EPQ = Eysenck Personality Questionnaire-Revised short form. DMSC = Dual-Modes of Self-Control Scale. *** *p* < 0.001

On family functioning (FACES II), mANOVA models revealed that there were significant group differences in both family adaptability (Time 1: *F*_(2, 2517)_ = 169.591, *p* < 0.001, *η*_p_^2^ = 0.119; Time 2: *F*_(2, 2517)_ = 121.522, *p* < 0.001, *η*_p_^2^ = 0.088) and family cohesion (Time 1: *F*_(2, 2517)_ = 168.547, *p* < 0.001, *η*_p_^2^ = 0.118; Time 2: *F*_(2, 2517)_ = 135.568, *p* < 0.001, *η*_p_^2^ = 0.097) across the two time points. Post-hoc tests showed that the no NSSI group had higher family adaptability and family cohesion than the emerging NSSI (Time 1 family adaptability: Cohen’s *d* = 0.30, *p* = 0.004; Time 2 family adaptability: Cohen’s *d* = 0.30, *p* = 0.005; Time 1 family cohesion: Cohen’s *d* = 0.30, *p* < 0.001; Time 2 family cohesion: Cohen’s *d* = 0.24, *p* = 0.015) and sustained NSSI (Time 1 family adaptability: Cohen’s *d* = 0.90, *p* < 0.001; Time 2 family adaptability: Cohen’s *d* = 0.78, *p* < 0.001; Time 1 family cohesion: Cohen’s *d* = 0.88, *p* < 0.001; Time 2 family cohesion: Cohen’s *d* = 0.80, *p* < 0.001) groups, and the emerging NSSI group scored higher than the sustained NSSI group both on family adaptability (Time 1: Cohen’s *d* = 0.58, *p* < 0.001; Time 2: Cohen’s *d* = 0.47, *p* < 0.001) and family cohesion (Time 1: Cohen’s *d* = 0.57, *p* < 0.001; Time 2: Cohen’s *d* = 0.57, *p* < 0.001).

On perceived school climate (SCS), there were significant group differences in teacher support (Time 1: *F*_(2, 2517)_ = 25.155, *p* < 0.001, *η*_p_^2^ = 0.020; Time 2: *F*_(2, 2517)_ = 18.160, *p* < 0.001, *η*_p_^2^ = 0.014) and peer support (Time 1: *F*_(2, 2517)_ = 81.323, *p* < 0.001, *η*_p_^2^ = 0.061; Time 2: *F*_(2, 2517)_ = 49.592, *p* < 0.001, *η*_p_^2^ = 0.038), but not in autonomy opportunities (Time 1: *F*_(2, 2517)_ = 0.261, *p =* 0.770; Time 2: *F*_(2, 2517)_ = 0.099, *p* = 0.906). Post-hoc tests showed that the no NSSI group did not differ from the emerging NSSI group in teacher support or peer support (all *p* > 0.05), but these two groups scored higher than the sustained NSSI group on teacher support (Cohen’s *d* = 0.22–0.40, all *p* < 0.001) and peer support (Cohen’s *d* = 0.42–0.64, all *p* < 0.001) across the time points.

On negative life events (ASLEC), significant between-group differences were found (Time 1: *F*_(2, 2517)_ = 495.744, *p* < 0.001, *η*_p_^2^ = 0.283; Time 2: *F*_(2, 2517)_ = 321.596, *p* < 0.001, *η*_p_^2^ = 0.204). Post-hoc tests showed that compared with the no NSSI group, the emerging NSSI group had more negative life events just in Time 2 (Cohen’s *d* = 0.80, *p* < 0.001) but not in Time 1 (*p* = 0.941), while the sustained NSSI group scored higher in both time points (Time 1: Cohen’s *d* = 1.36, *p* < 0.001; Time 2: Cohen’s *d* = 1.19, *p* < 0.001). The sustained NSSI group had more life events than the emerging NSSI group (Time 1: Cohen’s *d* = 1.21, *p* < 0.001; Time 2: Cohen’s *d* = 0.33, *p* < 0.001).

On personality trait (EPQ), significant group differences were displayed in neuroticism (Time 1: *F*_(2, 2517)_ = 829.115, *p* < 0.001, *η*_p_^2^ = 0.397; Time 2: *F*_(2, 2517)_ = 779.662, *p* < 0.001, *η*_p_^2^ = 0.383). Post-hoc tests showed that compared to the no NSSI group, the emerging NSSI (Time 1: Cohen’s *d* = 0.88, *p* < 0.001; Time 2: Cohen’s *d* = 0.66, *p* < 0.001) and sustained NSSI (Time 1: Cohen’s *d* = 1.92, *p* < 0.001; Time 2: Cohen’s *d* = 1.89, *p* < 0.001) groups had elevated levels of neuroticism. Besides, the sustained NSSI group scored higher on neuroticism than the emerging NSSI group across the time points (Time 1: Cohen’s *d* = 0.86, *p* < 0.001; Time 2: Cohen’s *d* = 1.10, *p* < 0.001).

On dual-system processes (DMSC), there were significant group differences in both impulse system (Time 1: *F*_(2, 2517)_ = 603.270, *p* < 0.001, *η*_p_^2^ = 0.324; Time 2: *F*_(2, 2517)_ = 565.619, *p* < 0.001, *η*_p_^2^ = 0.310) and self-control system (Time 1: *F*_(2, 2517)_ = 527.012, *p* < 0.001, *η*_p_^2^ = 0.295; Time 2: *F*_(2, 2517)_ = 430.900, *p* < 0.001, *η*_p_^2^ = 0.255). Post-hoc tests showed that compared with the no NSSI group, both the emerging NSSI (Time 1: Cohen’s *d* = 0.69, *p* < 0.001; Time 2: Cohen’s *d* = 0.45, *p* < 0.001) and sustained NSSI (Time 1: Cohen’s *d* = 1.61, *p* < 0.001; Time 2: Cohen’s *d* = 1.52, *p* < 0.001) groups scored higher on impulse system across the time points. Conversely, both the emerging NSSI (Time 1: Cohen’s *d* = 0.37, *p* = 0.001; Time 2: Cohen’s *d* = 0.35, *p* = 0.006) and sustained NSSI (Time 1: Cohen’s *d* = 1.48, *p* < 0.001; Time 2: Cohen’s *d* = 1.33, *p* < 0.001) groups scored lower on self-control system than the no NSSI group. The emerging NSSI group showed a lower impulse system score but a higher self-control system score than the sustained NSSI group across time points (Cohen’s *d* = 0.84–1.10, all *p* < 0.001).

### Partial correlations between psychological variables and NSSI scores

The partial correlations showed that the NSSI scores (Time 2) were positively correlated with total negative life events, neuroticism, and impulse system scores (*r*_p_=0.406–0.559, all *p* < 0.001), but negatively correlated with family adaptability, family cohesion, teacher support, peer support, and self-control system scores (*r*_p_= -0.120 to -0.508, all *p* < 0.001). However, there were no significant correlations between autonomy opportunities and the NSSI scores (Supplementary Table S3).

### Logistic regression outcomes

Two binary regression models, one for no NSSI group vs. emerging NSSI group and the other for no NSSI group vs. sustained NSSI group, were conducted using a 2-step design: age (Time 1), grade (Time 1), gender, ethnicity, single-child, left-behind child, home locality, and parents’ educational level were entered in step 1 as the control variables, and all psychological variables were entered in step 2. The outcomes were presented in Table 3. In the no NSSI vs. emerging NSSI model (*N* = 1841, *Nagelkerke R*^2^ = 0.343), Time 1 neuroticism scores (*OR* = 1.382, *p* < 0.001, 95% CI = 1.256–1.521) and Time 1 impulse system scores (*OR* = 1.189, *p* < 0.001, 95% CI = 1.123–1.259), as well as Time 2 negative life events (*OR* = 1.040, *p* < 0.001, 95% CI = 1.031–1.049) and Time 2 impulse system scores (*OR* = 1.145, *p* < 0.001, 95% CI = 1.089–1.197), positively predicted emerging NSSI behavior. In the no NSSI vs. sustained NSSI model (*N* = 2266, *Nagelkerke R*^2^ = 0.464), Time 1 neuroticism scores (*OR* = 1.313, *p* < 0.001, 95% CI = 1.200-1.435), Time 1 impulse system scores (*OR* = 1.060, *p* < 0.01, 95% CI = 1.022-1.100), Time 1 negative life events (*OR* = 1.043, *p* < 0.001, 95% CI = 1.029–1.057), Time 2 impulse system scores (*OR* = 1.012, *p* < 0.05, 95% CI = 1.000-1.024), and Time 2 negative life events (*OR* = 1.036, *p* < 0.001, 95% CI = 1.027–1.045) positively, while Time 1 self-control system scores (*OR* = 0.832, *p* < 0.001, 95% CI = 0.797–0.869) negatively, predicted sustained NSSI behavior.

**Table 3:** Logistic regression analyses of psychological variables on NSSI behaviors

Models	Emerging NSSI ^a^			Sustained NSSI ^b^		
	B	Wald χ^2^	OR (95% CI)	B	Wald χ^2^	OR (95% CI)
**Step 1**						
Age (T1)	-0.178	1.551	0.837 (0.633–1.108)	-0.361	7.890**	0.697 (0.542–0.897)
Grade (T1)	0.309	1.224	1.362 (0.788–2.354)	-0.974	17.985***	0.378 (0.241–0.592)
Gender	-0.164	0.982	0.849 (0.614–1.174)	0.318	4.637*	1.374 (1.029–1.836)
Ethnicity	-0.033	0.017	0.968 (0.597–1.570)	0.416	3.766	1.516 (0.996–2.309)
Single-child	0.325	1.780	1.384 (0.859–2.231)	-0.306	2.075	0.736 (0.485–1.117)
Left-behind child	-0.280	2.890	0.756 (0.547–1.044)	-0.175	1.360	0.839 (0.625–1.127)
Home locality	0.813	17.463***	2.254 (1.540–3.300)	0.567	12.150***	1.433 (1.220–1.588)
Education level of father	-0.423	1.856	0.655 (0.375–1.204)	-0.565	3.463	0.568 (0.313–1.031)
Education level of mother	-0.143	0.295	0.867 (0.471–1.595)	-0.320	1.017	0.726 (0.390–1.352)
**Step 2**						
Family adaptability (T1)	0.024	1.308	1.025 (0.983–1.068)	-0.034	3.289	0.966 (0.931–1.003)
Family adaptability (T2)	-0.022	1.560	0.979 (0.946–1.012)	0.001	0.001	1.001 (0.969–1.032)
Family cohesion (T1)	-0.055	2.765	0.946 (0.895–1.002)	-0.020	1.494	0.981 (0.950–1.012)
Family cohesion (T2)	0.039	2.195	1.039 (0.988–1.094)	0.018	1.618	1.018 (0.990–1.046)
Teacher support (T1)	0.051	1.443	1.052 (0.968–1.143)	-0.032	0.181	0.969 (0.837–1.121)
Teacher support (T2)	-0.047	1.289	0.954 (0.879–1.035)	0.046	0.384	1.047 (0.906–1.210)
Peer support (T1)	-0.005	0.030	0.995 (0.943–1.050)	-0.038	1.202	0.963 (0.899–1.030)
Peer support (T2)	0.018	0.574	1.018 (0.972–1.066)	0.023	0.531	1.023 (0.962–1.089)
Autonomy opportunities (T1)	0.102	0.113	1.108 (0.610–2.011)	0.059	1.824	1.060 (0.974–1.154)
Autonomy opportunities (T2)	-0.041	0.016	0.959 (0.502–1.832)	0.061	2.156	1.063 (0.980–1.154)
Total life events (T1)	0.018	0.928	1.018 (0.982–1.055)	0.044	33.856***	1.043 (1.029–1.057)
Total life events (T2)	0.039	79.387***	1.040 (1.031–1.049)	0.035	60.002***	1.036 (1.027–1.045)
Neuroticism (T1)	0.324	43.728***	1.382 (1.256–1.521)	0.272	35.593***	1.313 (1.200-1.435)
Neuroticism (T2)	-0.097	3.749	0.908 (0.823–1.001)	0.037	0.678	1.038 (0.950–1.134)
Impulse system (T1)	0.173	35.403***	1.189 (1.123–1.259)	0.059	9.766**	1.060 (1.022-1.100)
Impulse system (T2)	0.156	23.554***	1.145 (1.089–1.197)	0.012	3.710*	1.012 (1.000-1.024)
Self-control system (T1)	-0.035	1.197	0.965 (0.906–1.028)	-0.183	68.967***	0.832 (0.797–0.869)
Self-control system (T2)	-0.002	0.002	0.998 (0.936–1.065)	-0.031	1.984	0.969 (0.928–1.012)

Note: NSSI = Non-suicidal self-injury. No NSSI = the subgroup of individuals without NSSI (i.e., who were negative in both time points). Emerging NSSI = the subgroup of individuals who were negative in Time 1 but positive in Time 2. Sustained NSSI = the subgroup of individuals who were positive in both time points. T1 = Time 1 (Sep. 2019), T2 = Time 2 (Sep. 2020). CI = confidence interval. OR = odds ratio

^a^ No NSSI (coded as 0) vs. Emerging NSSI (coded as 1) model (*N* = 1841), Nagelkerke *R*^2^ = 0.343

^b^ No NSSI (coded as 0) vs. Sustained NSSI (coded as 1) model (*N* = 2266), Nagelkerke *R*^2^ = 0.464

* *p* < 0.05, ** *p* < 0.01, *** *p* < 0.001

For the purpose of detecting psychological variables in the development of NSSI from emerging NSSI to sustained NSSI behaviors, we also conducted another binary regression model (emerging NSSI group vs. sustained NSSI group) with the similar 2-step design (Table 4). In this model (*N* = 947, *Nagelkerke R*^2^ = 0.468), Time 1 negative life events (*OR* = 1.066, *p* < 0.001, 95% CI = 1.050–1.081), Time 1 neuroticism levels (*OR* = 1.214, *p* < 0.001, 95% CI = 1.106–1.332), Time 1 impulse-system scores (*OR* = 1.098, *p* < 0.001, 95% CI = 1.063–1.135), and Time 2 impulse-system scores (*OR* = 1.053, *p* < 0.01, 95% CI = 1.024–1.083) positively, while Time 1 self-control system scores (*OR* = 0.883, *p* < 0.001, 95% CI = 0.832–0.936) negatively, predicted sustained NSSI behavior when emerging NSSI behavior was set as the reference.

**Table 4:** Psychological variables in the emerging NSSI *versus* sustained NSSI model

Models	Emerging NSSI (0) vs. Sustained NSSI (1) ^a^		
	B	Wald χ^2^	OR (95% CI)
**Step 1**			
Age (T1)	0.009	0.005	1.009 (0.787–1.293)
Grade (T1)	-0.464	5.045*	0.628 (0.419–0.943)
Gender	0.019	0.015	1.019 (0.759–1.367)
Ethnicity	0.199	0.789	1.220 (0.787–1.891)
Single-child	0.187	0.595	1.205 (0.750–1.937)
Left-behind child	-0.260	2.928	0.771 (0.572–1.039)
Home locality	-0.028	0.236	0.921 (0.662–1.282)
Education level of father	0.098	0.106	1.103 (0.612–1.989)
Education level of mother	0.594	3.551	1.810 (0.976–3.357)
**Step 2**			
Family adaptability (T1)	-0.039	2.723	0.962 (0.919–1.007)
Family adaptability (T2)	0.007	0.132	1.007 (0.970–1.045)
Family cohesion (T1)	0.011	0.263	1.011 (0.969–1.055)
Family cohesion (T2)	0.004	0.039	1.004 (0.964–1.045)
Teacher support (T1)	-0.009	0.040	0.991 (0.904–1.085)
Teacher support (T2)	0.016	0.117	1.017 (0.925–1.117)
Peer support (T1)	-0.066	3.063	0.936 (0.869–1.008)
Peer support (T2)	0.051	2.093	1.053 (0.982–1.129)
Autonomy opportunities (T1)	0.053	1.222	1.054 (0.960–1.158)
Autonomy opportunities (T2)	0.047	0.933	1.048 (0.953–1.152)
Total life events (T1)	0.064	73.891***	1.066 (1.050–1.081)
Total life events (T2)	-0.003	0.300	0.997 (0.988–1.007)
Neuroticism (T1)	0.194	16.755***	1.214 (1.106–1.332)
Neuroticism (T2)	-0.068	2.294	0.934 (0.856–1.020)
Impulse system (T1)	0.094	32.086***	1.098 (1.063–1.135)
Impulse system (T2)	0.048	9.204**	1.053 (1.024–1.083)
Self-control system (T1)	-0.125	17.354***	0.883 (0.832–0.936)
Self-control system (T2)	-0.026	0.741	0.974 (0.918–1.034)

Note: NSSI = Non-suicidal self-injury. Emerging NSSI = the subgroup of individuals who were negative in Time 1 but positive in Time 2. Sustained NSSI = the subgroup of individuals who were positive in both time points. T1 = Time 1 (Sep. 2019), T2 = Time 2 (Sep. 2020). CI = confidence interval. OR = odds ratio. ^a^ Logistic regression model for Emerging NSSI (coded as 0) vs. Sustained NSSI (coded as 1), *N* = 947, Nagelkerke *R*^2^ = 0.468. * *p* < 0.05, ** *p* < 0.01, *** *p* < 0.001

## Discussion

This follow-up study demonstrated the aggravated impact of the COVID-19 pandemic on NSSI in high school students, and indicated that higher neuroticism, elevated impulsivity, and lower self-control traits could be the key risk factors for the occurrence and maintenance of adolescent NSSI behavior in the pandemic context.

### Prevalence of NSSI in high school students before and during the COVID-19

The prevalence of NSSI in Chinese high school students has varied in previous studies from 6.80 to 37.1% [18, 19, 80, 81]. In western cultures, the incidence rate of NSSI among high school students ranged from 13.0 to 32.2% [82–86]. In our study, the prevalence rate of NSSI was 27.2% before the COVID-19 pandemic, and increased by 10.3% during the pandemic. The variations might be due to different samples, methodologies, time points, as well as the classification criteria for NSSI. Therefore, universal criteria and measures for NSSI should be used in future studies. Nevertheless, our data indicated a direct pandemic effect on adolescent mental health, suggesting a deterioration in NSSI due to the pandemic among high school students. More longitudinal research from before, during to after the COVID-19 pandemic on mental health for students should be helpful for future public health strategies [7, 8].

### Psychological risk variables linked to NSSI behavior

In this study, we firstly classified the subjects into no NSSI, emerging NSSI, and sustained NSSI subgroups according to their NSSI behaviors before and during the pandemic, and compared the perceived family functioning and school climate, negative life events, and individual traits (neuroticism, impulsivity, and self-control) at two time points (i.e., Time 1 and Time 2) between the three subgroups (Table 2). Then logistic regression models were used to test the predictive effects of these psychological/social variables on emerging and sustained NSSI behaviors (Table 3).

For perceived family functioning, mANOVA models revealed lower levels of family adaptability and family cohesion in the emerging NSSI subgroup with a small effect size (Cohen’s *d* = 0.24 to 0.30) and in the sustained NSSI subgroup with a large effect size (Cohen’s *d* = 0.78 to 0.90), compared to the no NSSI subgroup. Besides, NSSI scores were negatively correlated with family adaptability and cohesion scores.

These data further supported previous research findings that poor family functioning were associated with NSSI in adolescents [48–50]. It has been assumed that families with high-quality functioning may have high levels of parent-child involvement and adapt better in the face of conflicts, which would consequently prevent adolescents from engaging in negative and anti-social behaviors [87]. Indeed, family dysfunction was indicated as a robust risk factor for NSSI among adolescents [51, 52]. However, our study did not find a significant predictive effect of reduced family functioning on emerging or sustained NSSI behavior in the logistic regressions. Possible mediating and/or moderating variables might play a part between family functioning and NSSI [36], and more efforts should be made to verify their relationships in the adolescents.

For perceived school climate, our data showed that the emerging NSSI subjects scored similarly with the no NSSI subgroup on teacher support, peer support, and autonomy opportunities, while the sustained NSSI subgroup had lower scores than the no NSSI subgroup on teacher support and peer support with a moderate to large effect size (Cohen’s *d =* 0.40–0.64). Our data were consistent with previous studies displaying negative associations of teachers’ support and peer climate with NSSI in high school students [41, 53]. However, there were no any predictive effects of these facets of perceived school climate (i.e., teacher support and peer support) on NSSI behavior in our study. Previously, it has been supposed that students might be less likely to engage in NSSI within a positive school climate [41]. However, further studies are needed to detect the role of teacher/peer support in NSSI at great length.

Life stress is a well-recognized risk factor that takes effect in many psychiatric and psychological disorders [88, 89]. In accordance with previous research [43, 57, 58], our study indicated positive relationships between accumulated negative life events and NSSI. Especially, the sustained NSSI individuals reported to experience more life events before and during the COVID-19 pandemic than no NSSI subgroup. Interestingly, the emerging NSSI subjects merely reported more negative life events during (but not before) the pandemic, compared to the no NSSI subgroup. Therefore,

a higher level of perceived life stress due to the COVID-19 pandemic might be an important factor for susceptible individuals to be involved into NSSI behaviors [59]. The assumption that life stress should be a probable contributor to NSSI was further supported by our logistic regression outcomes, showing that total negative life events positively predicted both emerging NSSI and sustained NSSI behaviors. Furthermore, we also found that more negative life events experienced before the pandemic could positively predict a possible transition from emerging NSSI behavior to sustained NSSI behavior (Table 4). These findings highlighted the harmful role of cumulative life stress in the development of NSSI for adolescent students, particularly for those who possess potential vulnerabilities, and more attention should be paid to this issue.

Some typical personality traits, such as impulsivity and neuroticism, have often been considered the individual dispositional factors in the development of disordered behaviors including addiction and suicide [90, 91]. Indeed, high-level neuroticism and impulsivity were found in adolescent and college students with NSSI [64–69]. In essence, neuroticism represents emotional instability or capriciousness. Individuals with higher neuroticism could react strongly to even a little negative change [92]. In our study, both the emerging NSSI and sustained NSSI individuals showed elevated levels of neuroticism than the no NSSI subgroup, with a large effect size across the two time points (Cohen’s *d* = 0.66–1.92). In addition, neuroticism before the pandemic was indicated as a positive predictor for both emerging and sustained NSSI during the pandemic, supporting previous reports that manifested neuroticism an important predictor for adolescent NSSI [67]. Similarly, heightened impulsivity (i.e., impulse system) was closely linked to NSSI, consistent with previous findings [34, 68], and Time 1 impulsivity levels positively predicted emerging NSSI and sustained NSSI behaviors in our study. Interestingly, we detected lower self-control (i.e., self-control system) in both the emerging NSSI (Cohen’s *d =* 0.35–0.37) and sustained NSSI (Cohen’s *d =* 1.33–1.48) subgroups compared with the no NSSI subgroup. However, self-control was a negative predictor just for sustained NSSI but not for emerging NSSI, and lower self-control scores (Time 1) predicted sustained NSSI behavior when emerging NSSI behavior was set as the reference (Table 4). These data added to previous reports that poor self-control was a risk factor for adolescent NSSI [70–72], revealing an important role of deficient self-control in the maintenance and development of NSSI behavior among the adolescent students. Nevertheless, the effects of self-control implicated in the occurrence of NSSI (i.e., emerging NSSI) during the pandemic should be further studied with a longer period. Remarkably, recent imaging studies have elucidated top-down neural alterations related to the regulatory systems (e.g., reduced anterior cingulate cortex volume) in NSSI for youths [93], which might underline the possible neurobiological bases of impulsivity and self-control linked to NSSI. Thus, further studies are warranted on this issue.

### Study limitations

Several limitations in the present study should also be noted. Firstly, the period of our follow-up investigation was only one year from before to during the COVID-19 pandemic. In addition, the pandemic is far from completely over by now, influencing the economics, society, and the daily life, at least in China. Thus, the future follow-up studies on adolescent mental health development after the pandemic are still needed. Secondly, this study was conducted in a provincial population of high school students, as a result it might not be a nationally representative sample. It is unclear whether our results could be generalized into a broader range of adolescent populations or in other cultures. Similar cross-cultural studies should be of great help to confirm the findings. Thirdly, self-report measures were mainly used in the study, thus potential subjective bias from the participants might not be effectively avoided, and our findings should be interpreted and used carefully. Hence, more objective tools such as laboratory-based tasks are warranted to achieve more conclusive data and results in future research.

### Conclusions and implications

In despite of the limitations, our findings in this study depicted the aggravated impact of the COVID-19 pandemic on adolescent NSSI in a one-year follow-up, and added to the current evidence for understanding the psychological contributors to the development of NSSI among adolescent students, revealing that higher neuroticism and impulsivity, and lower self-control were linked to emerging and sustained NSSI.

As many studies pointed out, the COVID-19 pandemic has caused significant disruptions to the lives and negative impacts on the mental health of children and adolescents around the world [94, 95], and increasing need for research, monitoring, prevention, and intervention for mental health of children and adolescents remains urgent during the current and future pandemics [94–96]. Our findings in the present study thus might be beneficial for developing potential prevention and intervention methods for NSSI behaviors of adolescent students. For one thing, the COVID-19 pandemic and its associated infection control measures (e.g., school closures, social lockdown) suddenly magnified the perceived life stress among the students, which might increase the risk of being involved into NSSI behaviors for them. Our follow- up data indicated an increase (10.3%) in the incidence of NSSI during the pandemic. Therefore, at the policy/system level, school-based health centers and mental health services would be necessary to support students’ mental health and well-being [96]. For instance, mobile mental health units might represent an important access for the students and their families in terms of an emergency stress coping to the pandemic. Online behavioral training and contingency management techniques that are aimed at coping and regulating stress-related negative affects might thus help to reduce inappropriate catharsis of stress and prevent the impulsive NSSI behaviors (97–98). For another thing, our findings directly suggested that higher neuroticism, elevated impulsivity, and lower self-control could be the critical psychological contributors to the occurrence (emerging NSSI), continuation (sustained NSSI), and transition (from emerging to sustained NSSI) of NSSI behaviors among adolescent students. These individual personality traits probably constitute the “impulsive facets” of NSSI [72]. More importantly, higher neuroticism, elevated impulsivity, and lower self-control before the COVID-19 pandemic were indicated as the dispositional vulnerabilities that positively predicted the occurrence, continuation and transition of NSSI during the pandemic. As a result, school-based mental health centers should be particularly concerned about susceptible students with these vulnerabilities, not only during the pandemic but also in routine care, for supporting their health and well-being [96]. In this aspect, increased innovations and digital resilience on the coping strategies of mental health promotion for adolescent students might be an adaptive alternate for schools and education institutions over time from before to after the pandemic [99]. However, it is still a long way for us to find the best coping strategy to the pandemic.

## Electronic supplementary material

Below is the link to the electronic supplementary material.

Supplementary Material 1

## Data Availability

Data could be obtained by contacting the corresponding author.
