# Supplementary material for: Impact of the COVID-19 and psychological risk factors on non-suicidal self-injury behavior among high school students: a one-year follow-up study
Source: BMC Psychiatry. 2023 Jul 14;23:512. doi: 10.1186/s12888-023-05021-2 (PMC10349405; doi:10.1186/s12888-023-05021-2)
Supplement: Supplementary file 1 — Supplementary Material 1 [file 12888_2023_5021_MOESM1_ESM.doc]

**Supplementary Materials**

**Table S1.** Comparison on demographic characteristics of the two samples.

| **Variables** | **Time 1 (Sep. 2019)** | **Time 2 (Sep. 2020)** | ***t*/χ^2^** | ***p*** |
| --- | --- | --- | --- | --- |
| Number of subjects (*N*) | 3588 | 2527 | - |  |
| Years of age (M±SD) | 16.66*±*1.09 | 17.13*±*0.79 | -18.440*** | <0.001 |
| Gender, Boys n (%) | 1778 (49.6) | 1217 (48.2) | 1.153 | 0.283 |
| Ethnicity, Hans n (%) | 3166 (88.2) | 2220 (87.9) | 0.212 | 0.645 |
| Single-child, Yes n (%) | 554 (15.4) | 357 (14.1) | 2.016 | 0.156 |
| Left-behind child, Yes n (%) | 1380 (38.5) | 985 (39.0) | 0.167 | 0.682 |
| Home locality, Urban n (%) | 2004 (55.9) | 1361 (53.9) | 2.383 | 0.123 |
| Grade, n (%) |  |  | - |  |
| 10th Grade | 1281 (35.7) | - |  |  |
| 11th Grade | 1269 (35.4) | 1273 (50.4) |  |  |
| 12th Grade | 1038 (28.9) | 1254 (49.6) |  |  |
| Education level of father, n (%) |  |  | 1.977 | 0.372 |
| Junior high school and below | 2204 (61.4) | 1597 (63.2) |  |  |
| Senior high school | 1008 (28.1) | 678 (26.8) |  |  |
| College/university and above | 376 (10.5) | 252 (10.0) |  |  |
| Education level of mother, n (%) |  |  | 0.552 | 0.759 |
| Junior high school and below | 2482 (69.2) | 1770 (70.0) |  |  |
| Senior high school | 676 (18.8) | 460 (18.2) |  |  |
| College/university and above | 430 (12.0) | 297 (11.8) |  |  |
| Individuals with NSSI, n (%) | 876 (24.4) | 947 (37.5) | 120.873*** | <0.001 |
| NSSI (FASM) scores (M±SD) | 2.97*±*6.32 | 4.10*±*7.31 | -6.426*** | <0.001 |

Note: NSSI=Non-suicidal self-injury. FASM=the Functional Assessment of Self-Mutilation.

*** *p* < 0.001.

**Table S2.** Demographic characteristics of the follow-up sample (*N*=2527).

| **Variables** | **Time 1 (Sep. 2019)** | **Time 2** **(Sep. 2020)** | ***t*/χ^2^** | ***p*** |
| --- | --- | --- | --- | --- |
| Years of age (M±SD) | 16.13*±*0.79 | 17.13*±*0.79 | -44.725*** | <0.001 |
| Gender, Boys n (%) | 1217 (48.2) | 1217 (48.2) | - | - |
| Ethnicity, Hans n (%) | 2220 (87.9) | 2220 (87.9) | - | - |
| Single-child, Yes n (%) | 357 (14.1) | 357 (14.1) | - | - |
| Left-behind child, Yes n (%) | 985 (39.0) | 985 (39.0) | - | - |
| Home locality, Urban n (%) | 1361 (53.9) | 1361 (53.9) | - | - |
| Grade, n (%) |  |  | - | - |
| 10th Grade | 1273 (50.4) | - |  |  |
| 11th Grade | 1254 (49.6) | 1273 (50.4) |  |  |
| 12th Grade | - | 1254 (49.6) |  |  |
| Education level of father, n (%) |  |  | - | - |
| Junior high school and below | 1597 (63.2) | 1597 (63.2) |  |  |
| Senior high school | 678 (26.8) | 678 (26.8) |  |  |
| College/university and above | 252 (10.0) | 252 (10.0) |  |  |
| Education level of mother, n (%) |  |  | - | - |
| Junior high school and below | 1770 (70.0) | 1770 (70.0) |  |  |
| Senior high school | 460 (18.2) | 460 (18.2) |  |  |
| College/university and above | 297 (11.8) | 297 (11.8) |  |  |
| Individuals with NSSI, n (%) | 686 (27.2) | 947 (37.5) | 61.628*** | <0.001 |
| NSSI (FASM) scores (M±SD) | 3.15*±*6.11 | 4.10*±*7.31 | -4.981*** | <0.001 |

Note: NSSI=Non-suicidal self-injury. FASM=the Functional Assessment of Self-Mutilation.

*** *p*<0.001.

**Table S3. Partial correlations between psychological variables and NSSI/FASM scores (*N*=2527).**

| **Variables** | **1** | **2** | **3** | **4** | **5** | **6** | **7** | **8** | **9** | **10** | **11** | **12** | **13** | **14** | **15** | **16** | **17** | **18** |
| --- | --- | --- | --- | --- | --- | --- | --- | --- | --- | --- | --- | --- | --- | --- | --- | --- | --- | --- |
| 1. Family adaptability (T1) | - | 0.850*** | 0.805*** | 0.709*** | 0.270*** | 0.239*** | 0.251*** | 0.204*** | 0.177*** | 0.185*** | -0.320*** | -0.229*** | -0.377*** | -0.379*** | -0.390*** | -0.363*** | 0.413*** | 0.361*** |
| 2. Family adaptability (T2) | 0.850*** | - | 0.681*** | 0.603** | 0.227*** | 0.207*** | 0.212*** | 0.178*** | 0.161*** | 0.140*** | -0.281*** | -0.191*** | -0.319*** | -0.320*** | -0.343*** | -0.322*** | 0.358*** | 0.318*** |
| 3. Family cohesion (T1) | 0.805*** | 0.681*** | - | 0.864*** | 0.302*** | 0.265*** | 0.297*** | 0.242*** | 0.163*** | 0.171*** | -0.341*** | -0.238*** | -0.382*** | -0.393*** | -0.380*** | -0.358*** | 0.409*** | 0.355*** |
| 4. Family cohesion (T2) | 0.709*** | 0.603** | 0.864*** | - | 0.268*** | 0.246*** | 0.272*** | 0.216*** | 0.126*** | 0.140*** | -0.328*** | -0.236*** | -0.350*** | -0.361*** | -0.356*** | -0.338*** | 0.391*** | 0.346*** |
| 5. Teacher support (T1) | 0.270*** | 0.227*** | 0.302*** | 0.268*** | - | 0.886*** | 0.492*** | 0.400*** | 0.457*** | 0.479*** | -0.217*** | -0.137*** | -0.200*** | -0.216*** | -0.155*** | -0.154*** | 0.191*** | 0.167*** |
| 6. Teacher support (T2) | 0.239*** | 0.207*** | 0.265*** | 0.246*** | 0.886*** | - | 0.452*** | 0.359*** | 0.422*** | 0.452*** | -0.191*** | -0.123*** | -0.174*** | -0.186*** | -0.138*** | -0.130*** | 0.164*** | 0.146*** |
| 7. Peer support (T1) | 0.251*** | 0.212*** | 0.297*** | 0.272*** | 0.492*** | 0.452*** | - | 0.823*** | 0.280*** | 0.259*** | -0.334*** | -0.222*** | -0.300*** | -0.313*** | -0.235*** | -0.237*** | 0.219*** | 0.196*** |
| 8. Peer support (T2) | 0.204*** | 0.178*** | 0.242*** | 0.216*** | 0.400*** | 0.359*** | 0.823*** | - | 0.211*** | 0.192*** | -0.269*** | -0.177*** | -0.247*** | -0.263*** | -0.194*** | -0.193*** | 0.168*** | 0.152*** |
| 9. Autonomy opportunities (T1) | 0.177*** | 0.161*** | 0.163*** | 0.126*** | 0.457*** | 0.422*** | 0.280*** | 0.211*** | - | 0.792*** | -0.061** | -0.049* | -0.061** | -0.084*** | -0.055** | -0.063** | 0.096*** | 0.083*** |
| 10. Autonomy opportunities (T2) | 0.185*** | 0.140*** | 0.171*** | 0.140*** | 0.479*** | 0.452*** | 0.259*** | 0.192*** | 0.792*** | - | -0.081*** | -0.049* | -0.073*** | -0.091*** | -0.057** | -0.050* | 0.106*** | 0.080*** |
| 11. Total life events (T1) | -0.320*** | -0.281*** | -0.341*** | -0.328*** | -0.217*** | -0.191*** | -0.334*** | -0.269*** | -0.061** | -0.081*** | - | 0.558*** | 0.573*** | 0.583*** | 0.516*** | 0.486*** | -0.432*** | -0.389*** |
| 12. Total life events (T2) | -0.229*** | -0.191*** | -0.238*** | -0.236*** | -0.137*** | -0.123*** | -0.222*** | -0.177*** | -0.049* | -0.049* | 0.558*** | - | 0.436*** | 0.450*** | 0.378*** | 0.349*** | -0.311*** | -0.277*** |
| 13. Neuroticism (T1) | -0.377*** | -0.319*** | -0.382*** | -0.350*** | -0.200*** | -0.174*** | -0.300*** | -0.247*** | -0.061** | -0.073*** | 0.573*** | 0.436*** | - | 0.898*** | 0.652*** | 0.614*** | -0.581*** | -0.536*** |
| 14. Neuroticism (T2) | -0.379*** | -0.320*** | -0.393*** | -0.361*** | -0.216*** | -0.186*** | -0.313*** | -0.263*** | -0.084*** | -0.091*** | 0.583*** | 0.450*** | 0.898*** | - | 0.655*** | 0.631*** | -0.592*** | -0.543*** |
| 15. Impulse system (T1) | -0.390*** | -0.343*** | -0.380*** | -0.356*** | -0.155*** | -0.138*** | -0.235*** | -0.194*** | -0.055** | -0.057** | 0.516*** | 0.378*** | 0.652*** | 0.655*** | - | 0.913*** | -0.729*** | -0.666*** |
| 16. Impulse system (T2) | -0.363*** | -0.322*** | -0.358*** | -0.338*** | -0.154*** | -0.130*** | -0.237*** | -0.193*** | -0.063** | -0.050* | 0.486*** | 0.349*** | 0.614*** | 0.631*** | 0.913*** | - | -0.695*** | -0.645*** |
| 17. Self-control system (T1) | 0.413*** | 0.358*** | 0.409*** | 0.391*** | 0.191*** | 0.164*** | 0.219*** | 0.168*** | 0.096*** | 0.106*** | -0.432*** | -0.311*** | -0.581*** | -0.592*** | -0.729*** | -0.695*** | - | 0.898*** |
| 18. Self-control system (T2) | 0.361*** | 0.318*** | 0.355*** | 0.346*** | 0.167*** | 0.146*** | 0.196*** | 0.152*** | 0.083*** | 0.080*** | -0.389*** | -0.277*** | -0.536*** | -0.543*** | -0.666*** | -0.645*** | 0.898*** | - |
| **19. T2 NSSI/FASM scores** | **-0.303***** | **-0.268***** | **-0.301***** | **-0.289***** | **-0.145***** | **-0.120***** | **-0.234***** | **-0.189***** | **-0.005** | **-0.003** | **0.484***** | **0.406***** | **0.559***** | **0.558***** | **0.550***** | **0.525***** | **-0.508***** | **-0.439***** |

Note: NSSI=Non-suicidal self-injury. FASM=the Functional Assessment of Self-Mutilation. T1=Time 1 (Sep. 2019), T2=Time 2 (Sep. 2020).

Control variables: Age (T1), Grade (T1), Gender, Ethnicity, Single-Child, Left-Behind Child, Home locality, Education level of father, and Education level of mother.

* *p* < 0.05, ** *p* < 0.01, *** *p* < 0.001.
